# 

**Published:** 1908-01

**Authors:** 


### BOOKS RECEIVED.

Mt. Sinai Hospital Reports. Vol. V. For 1905 and 1906. Edited by N. E. Brill, M.D.

Syphilis in its Medical, Medico-Legal and Sociological Aspects. By A. Ravogli, M.D., Professor of Dermatology and Syphilology in the Medical College of Ohio, Medical Department of Cincinnati University. Octavo, pp. 518. Illustrated. New York: The Grafton Press.

---

The Sexual Instinct. Its Use and Dangers as Affecting Heredity and Morals. By James Foster Scott, A.B., M.D., late Obstetrician to Columbia Hospital for Women, and Lying-in Asylum, Washington, D.C. Second edition. Small 8vo, p. 474. Illustrated. New York: E. B. Treat & Co. (Cloth, $2.00.)

---

Diseases of Children for Nurses. By Robert S. McCombs, M.D., 12mo, p. 431. Illustrated. Philadelphia and London: W. B. Saunders Co. (Cloth, $2.00.)

---

A Reference Handbook of Obstetric Nursing. By W. Reynolds Wilson, M.D., Visiting Physician to the Philadelphia Lying-in Charity. 12mo, pp. 258. Illustrated. Philadelphia and London: W. B. Saunders Company. (Limp leather, $1.75.)

---

Report of the Commissioner of Education for the year ending June 30, 1906. Volume I. Washington: Government Printing Office.

---

Thirty-fourth Annual Report of the State Board of Health of the State of Michigan for the fiscal year ending June 30, 1906. Frank W. Shumway, M.D., Secretary.

---

Modern Medicine. Its Theory and Practice. In original contributions by American and Foreign authors. Edited by William Osler, M.D., Regius Professor of Medicine in Oxford University, Eng. Assisted by Thomas McCrea, M.D., Associate Professor of Medicine and Clinical Therapeutics in Johns Hopkins University, Baltimore. In seven octavo volumes of about 900 pages each, illustrated. Volume III. Lea Brothers & Co., Philadelphia and New York, 1907. (Cloth, $6.00; leather, $7.00; half morocco, $7.50 net prices.)

---

Progressive Medicine, Vol. IX, December, 1907. A Quarterly Digest of Advances, Discoveries and Improvements in the Medical and Surgical Sciences. Edited by Hobart Amory Hare, M.D., Professor of Therapeutics and Materia Medica in the Jefferson Medical College of Philadelphia. Lea Brothers & Co., Philadelphia and New York. (Per annum, in four cloth-bound volumes, $9.00; in paper binding, $6.00, carriage paid to any address.)

---

The Elements of Homeopathic Theory, Materia Medica, Practice and Pharmacy. Compiled by Dr. F. A. Boericke and E. P. Anshutz. Second edition. 218 pages. Philadelphia. Boericke & Tafel. 1907. (Cloth, $1.00 net; postage 5 cents.)

